# EEG Microstates Analysis in Young Adults With Autism Spectrum Disorder During Resting-State

**DOI:** 10.3389/fnhum.2019.00173

**Published:** 2019-06-12

**Authors:** David F. D’Croz-Baron, Mary Baker, Christoph M. Michel, Tanja Karp

**Affiliations:** ^1^Autumn’s Dawn Neuroimaging, Cognition, and Engineering Laboratory, Department of Electrical and Computer Engineering, Texas Tech University, Lubbock, TX, United States; ^2^Functional Brain Mapping Laboratory, Department of Basic Neuroscience, Faculty of Medicine, University of Geneva, Geneva, Switzerland; ^3^Department of Electrical and Computer Engineering, Texas Tech University, Lubbock, TX, United States

**Keywords:** EEG microstates, autism spectrum disorder, resting state, topographical analysis, electroencephalography

## Abstract

Electroencephalography (EEG) is a useful tool to inspect the brain activity in resting state and allows to characterize spontaneous brain activity that is not detected when a subject is cognitively engaged. Moreover, taking advantage of the high time resolution in EEG, it is possible to perform fast topographical reference-free analysis, since different scalp potential fields correspond to changes in the underlying sources within the brain. In this study, the spontaneous EEG resting state (eyes closed) was compared between 10 young adults ages 18–30 years with autism spectrum disorder (ASD) and 13 neurotypical controls. A microstate analysis was applied, focusing on four temporal parameters: mean duration, the frequency of occurrence, the ratio of time coverage, and the global explained variance (GEV). Using data that were acquired from a 65-channel EEG system, six resting-state topographies that best describe the dataset across all subjects were identified by running a two-step cluster analysis labeled as microstate classes A–F. The results indicated that microstates B and E displayed statistically significant differences between both groups among the temporal parameters evaluated. Classes B, D, E, and F were consistently more present in ASD, and class C in controls. The combination of these findings with the putative functional significance of the different classes suggests that during resting state, the ASD group was more focused on visual scene reconstruction, while the control group was more engaged with self-memory retrieval. Furthermore, from a connectivity perspective, the resting-state networks that have been previously associated with each microstate class overlap the brain regions implicated in impaired social communication and repetitive behaviors that characterize ASD.

## Introduction

Autism spectrum disorder (ASD) is a developmental disorder that has an onset in early life and is characterized by repetitive behaviors, restricted interests, and impaired social communication (American Psychiatric Association, 2013). According to the United States Center for Disease Control and Prevention (CDC), the diagnosis of autism at age 2 is reliable, and about 1 in 59 children was diagnosed with ASD (Centers for Disease Control and Prevention, Autism Spectrum Disorder [ASD], 2018). Attempts to enhance social communication and maintain healthy and productive social interactions in individuals with ASD have motivated different studies, which assist in providing data that enables researchers to model the autistic brain (Billeci et al., 2013; Mash et al., 2018). Several approaches to evaluate and inspect the brain networks have been taken, including investigating resting-state and task-oriented electroencephalography (EEG).

Resting-state EEG is used to determine the brain activity when an explicit task is not being performed (Biswal, 2012); specifically, it may detect abnormalities that are not identified using evoked potentials (Fox, 2010; Wang et al., 2013). In the present study, the resting-state spontaneous EEG activity of ASD and neurotypical individuals (controls) young adults is analyzed.

EEG microstates analysis is a well-established technique used to study the resting state of the human brain based on the topography of the electric potentials over the electrode array. The principles of this method are described by Lehman and collaborators (Lehmann et al., 1987), who observed that a specific configuration of a global scalp map (or brain state), produced by the electric field measured via multichannel EEG, remains stable for around 80–120 ms and then switches to a new brain state belonging to a limited number of dominant quasi-stable scalp map set, which were defined considering only the electrode localization of the extreme potentials (maximal and minimal), ignoring polarity inversion. The spatial cluster was introduced in the microstate analysis by Pascual-Marqui et al. (1995), where the whole scalp topography (or scalp map) is considered, instead of using only the position of the extreme potentials as in (Lehmann et al., 1987). In this approach, a group of several scalp topographies with a high spatial correlation independent of polarity are clustered into one representative topography (template map, spatial map, or cluster) that best describes the variance within this group (Pascual-Marqui et al., 1995; Michel and Koenig, 2018). The microstates are then defined by fitting the set of template maps back to the temporal data also ignoring polarity inversion.

The microstate technique offers a collection of parameters with physiological relevance that have been widely used in the last 30 years to display variations across behavioral states, personality types, and neuropsychiatric disorders (Koenig et al., 2002; Khanna et al., 2015; Michel and Koenig, 2018), which make it a suitable tool to evaluate the dissimilarities in these parameters between ASD and control subjects. Indeed, a recent study (Jia and Yu, 2019) applied the microstate analysis among the two groups in resting state (combining eyes-open and eyes-closed conditions), finding significant differences and indicating that this technique may offer some intuitions into the intrinsic activities in the autistic brain. However, the limitations stated by the authors are the large age range, involving different periods of development such as middle childhood and adolescence, and a single analysis for the combined conditions. Therefore, the goal of the current study was to compare the EEG resting-state microstates (eyes-closed condition) between neurotypicals and ASD in the early adulthood, concentrating the analysis on the four well-established temporal parameters: (1) the mean duration, (2) frequency of occurrence, (3) the fraction of total time covered, and (4) the global explained variance (GEV).

## Materials and Methods

### Participants and Data Acquisition

The data set used in this analysis was obtained in a previous study. The experimental design and procedures, recording techniques, and participant data are described in more detail in Hames et al. (2016). Briefly, the EEG study had the participation of 16 neurotypical individuals (controls) and 15 autistic subjects (ASD) between the ages of 18 and 30 years. One subject in the ASD group is ambidextrous, and another from the same group is left-handed. The experiment was approved by the Human Subjects Internal Review Board at Texas Tech University, with written informed consent from all participants, in accordance with the Declaration of Helsinki. The study presented by Hames et al. (2016) consists of two sessions of different sensory tasks and one resting state (eyes closed). In this work, only the latter is considered for the EEG microstate analysis.

During the EEG resting-state recording, subjects were sitting in a comfortable upright position in a soundproof and electrical-shielded room. Participants were asked to stay as calm as possible, keeping their eyes closed for a time varying between 2 and 4 min. The EEG was acquired with a 65-channel monopolar EGI Hydrocel Geodesic Sensor using a sampling rate of 500 Hz (Electrical Geodesics Inc., Eugene, United States) with a vertex reference.

### EEG Data Processing

The preprocessing is carried out using a combination of MATLAB R2017b (The MathWorks) and the free academic software Cartool^1^ (Brunet et al., 2011). Microstate analysis is performed using only Cartool.

#### Preprocessing

The EEG data were band-pass filtered with half-power cutoff frequencies of 1 and 50 Hz applying a fourth-order non-causal Butterworth filter. The data were then visually inspected to detect and mark artifacts and bad epochs manually. Independent component analysis (ICA) was employed to identify and reject components associated with oculomotor activity and electrocardiography (ECG), as explained in Jung et al. (2000) and Makeig et al. (1996), corresponding to their waveform and topography. Only subjects with a number of samples greater than 20 times the number of channels squared (to obtain reliable decompositions) (Delorme and Makeig, 2004), after the visual inspection was performed, were considered in the ICA stage, reducing the number of participants to 13 neurotypicals and 10 ASD. The data were then down-sampled by a factor of 4 to 125 Hz to reduce computational time.

Cartool’s built-in spatial filtering function, which is based on the XYZ electrode coordinates (obtained from the manufacturer), was used to smooth the EEG signals and topographies for the posterior analysis. Finally, segments of ± 0.5 s around peaks with amplitudes above 100 μV, which are more associated with artifactual components rather than neural activity, were excluded from further analysis.

#### Microstate Analysis

**FIGURE 1:**
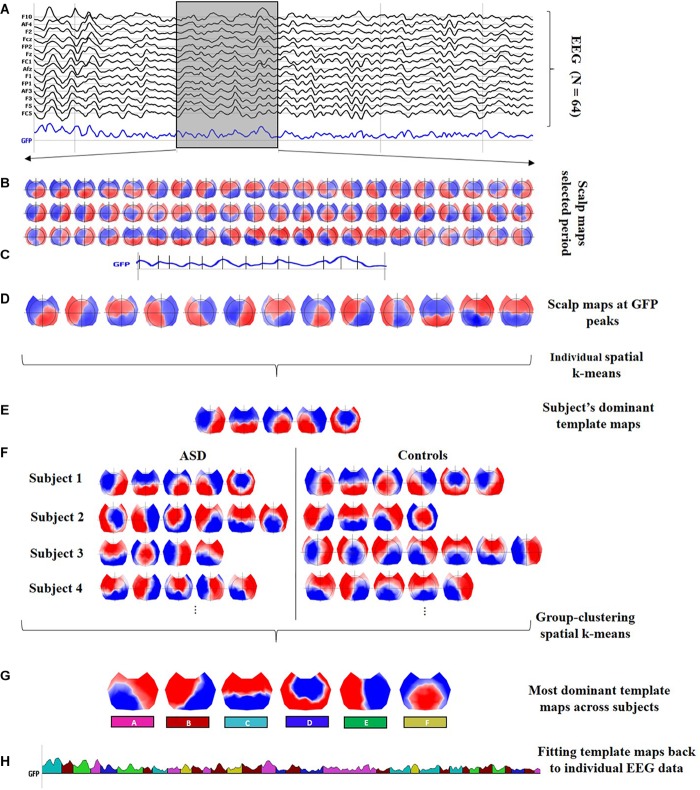
Microstate analysis. **(A)** Preprocessed EEG recordings down-sampled at 125 Hz, illustrating 2 s of data for one subject (vertical gray lines represent intervals of 0.5 s). Black curves correspond to 14 out of the *N* = 64 channels; the blue curve shows the global field power (GFP). Moreover, a 0.5-s interval is highlighted in the gray shaded area to display a zoom-in of the topographical data. **(B)** Sixty-three scalp maps from the 0.5-s interval, i.e., one per time frame. **(C)** Identification of the local peaks, displayed as vertical black lines, at the GFP curve within the 0.5-s interval. **(D)** The scalp maps corresponding to the local GFP peaks were submitted to a spatial k-means cluster analysis. **(E)** The most dominant template maps for the subject were selected based on the meta-criterion. **(F)** Steps **(A)** to **(E)** were repeated at individual level to obtain the set of the most dominant spatial maps for every subject. The individual sets with the dominant spatial maps for all subjects were submitted together to a group clustering analysis. **(G)** The six classes are the most dominant template maps after the group clustering spatial k-means across all subjects. The number of clusters was selected based on the meta-criterion. **(H)** A microstate sequence for the same subject as in **(A)**. The six classes are fitted back to the original EEG data of every subject, labeling each time frame with only one microstate, which is selected considering the highest spatial correlation between the scalp topography and every class (winner-takes-all). The microstate sequence is used, for every subject, to extract the temporal parameters and statistical analysis.

In general, microstate analysis consists of finding the set of the most dominant spatial maps (or template maps) from the different scalp topographies in the time domain, and then, the posterior labeling of the EEG data based on these dominant template maps. Therefore, after preprocessing the data, the stages involved in the microstate analysis are (1) segmentation of EEG data to find the most representative template maps, which correspond to the different classes, and (2) fitting these classes back to the EEG data to compute the temporal parameters and statistics. Figure 1 describes both stages with detailed steps.

In this work, the segmentation stage was carried out by running a two-step spatial cluster analysis, illustrated in Figure 1A–G, based on a modified version of the classical k-means algorithm (Pascual-Marqui et al., 1995), with the first step being run at the individual level (for each participant separately) (see Figure 1A–E) and the second step across all subjects (see Figure 1F,G; Murray et al., 2008; Tomescu et al., 2014; Gschwind et al., 2016). Although additional techniques are available to compute the segmentation stage (Poulsen et al., 2018; Von Wegner et al., 2018), a recent study reported that the underlying dynamics of the EEG signal seem to be independent of the initial clustering algorithm (Von Wegner et al., 2017, 2018).

To find each subject’s most dominant template maps, the global field power (GFP) was calculated for each sample time according to Equation (1), where *N* is the number of sensors in each scalp map, *u_i_* is the voltage at a specific electrode, and *ū* is the average voltage of the electrodes at the respective sample time.

(1) $$GFP=\sqrt{\frac{\sum_{i=1}^{N}{\left(u_{i}-\bar{u}\right)}^{2}}{N}}$$

The GFP is a reference-free measure that represents the field strength at a global level (Lehmann and Skrandies, 1980). The local peaks of the artifact-free GFP curve represent moments of high global neuronal synchronization (Skrandies, 2007) and the scalp topographies around them remain stable, maximizing the signal-to-noise ratio (SNR) (Koenig et al., 2002; Michel et al., 2009; Michel and Koenig, 2018). The scalp maps at sample times with a local GFP maximum (see Figure 1C) were submitted to a spatial k-means clustering algorithm to determine a subject’s most dominant template maps ignoring polarity inversion (see Figure 1E). The number of the dominant clusters was selected by a meta-criterion method described by Custo et al. (2017), which applies the information of seven different criteria from the literature.

To accomplish the second step in the two-step spatial cluster analysis, the dominant template maps for all subjects (ASD and controls) were submitted together to a spatial k-means group-cluster analysis to find the most representative maps across subjects, also denoted as classes. The number of classes was selected also ignoring polarity inversion based on the same meta-criterion as in step 1, resulting in the six microstate classes shown in Figure 1G.

Once the microstate classes were identified, they were fitted back to the individual EEG data in the temporal domain to define the microstates, as follows: each time frame (or sample point) of the original data was labeled with one microstate, considering the highest spatial correlation between the instantaneous scalp topography and every microstate class (winner-takes-all) (Murray et al., 2008; Michel and Koenig, 2018), but only if its correlation was above 0.5. In the fitting process, other temporal smoothing parameters such as strength 10 on a window half-size 3 [Besag factor λ = 10 and *b* = 3 in (Pascual-Marqui et al., 1995)] were applied to avoid interruptions in the EEG sequence associated with the same microstate. The microstate sequence is displayed color-coded in Figure 1H, and it is used, for every subject, to compute the different temporal parameters and the statistical analysis.

### Temporal Parameters and Statistical Analysis

The six microstate classes (A, B, C, D, E, and F) were computed considering the cluster analysis throughout all subjects to be able to compare the statistics between the ASD and controls, calculating the following temporal parameters for each class and every participant:

- Mean duration: This refers to the average duration, in milliseconds, that a microstate class is continuously presented.
- Frequency of occurrence: This indicates how often a microstate class is present per time interval and independent of the duration and is computed by taking the number of segments labeled with a microstate class divided by the total duration in seconds of the analyzed EEG.
- Fraction of time covered: This represents the proportion of the total time a microstate is present during the whole time considered for analysis.
- GEV: This parameter gives a metric of how well the selected template maps describe the whole dataset, calculated for a specific microstate class by summing the squared spatial correlations between the microstate specific template map and its corresponding labeled scalp maps at each time weighted by the GFP using Equation (2) (Murray et al., 2008) as follows: *GFP_u_* (*t*) is the GFP of the EEG data assigned to microstate class *u* at the labeled time *t*, and *C_u,T_t__* corresponds to the spatial correlation previously described.

(2) $$GEV_{u}=\frac{\sum_{t=1}^{t\;max}{\left(GFP_{u}(t)\cdot C_{u,\;T_{t}}\right)}^{2}}{\sum_{t=1}^{t\;max}\left(GFP_{u}^{2}(t)\right)}$$

The statistical analysis was performed using R version 3.4.3 (The R Foundation for Statistical Computing, 2017). Separate *post hoc* two-tailed Mann–Whitney–Wilcoxon tests were conducted pairwise between the two groups for each microstate class in every temporal parameter to identify statistically significant differences. Subsequently, the results were corrected for multiple comparisons by applying the false discovery rate (FDR), setting the significance at a 5% level (α = 0.05).

## Results

**FIGURE 2:**
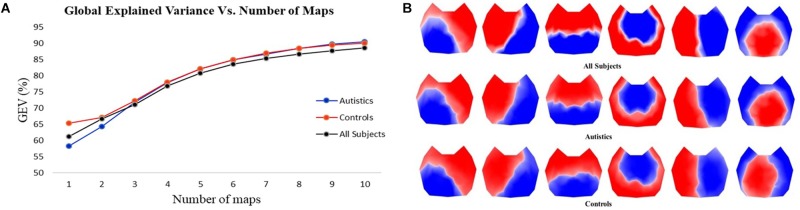
**(A)** GEV vs. number of template maps using three different approaches in the cluster analysis: considering only autistics (blue curve), only controls (red curve), and all subjects (black curve). **(B)** Template topographies of the six classes of microstates using three approaches: all subjects (top row), autistics (middle row), and controls (bottom row).

The first step in the two-step spatial cluster analysis identified between four and seven template maps, selected by the meta-criterion method, for each subject as illustrated in Figure 1F. The second step (group cluster) was firstly performed for each group separately to compare the different topographies. Based on the meta-criterion, the number of clusters that best described the dataset was seven for controls and six for ASD. Similarly, when the grand-clustering was run across all subjects, the best number of dominant maps was six. Figure 2A illustrates the GEV as a function of the number of maps when the group-cluster analysis was implemented only in autistics (blue curve), controls (red curve), and all subjects (black curve). As illustrated, considering the same number of maps, until eight, the GEV was slightly higher for the neurotypicals. Figure 2B depicts the six template maps that described more than 80% of the global variance in all three cases, following the same three approaches, i.e., all subjects (top row), only ASD (middle row), and controls (bottom row).

Since the resulting template maps showed high similarity regardless of the approach, the selected temporal parameters are computed using the same six topographies obtained from all subjects for both groups to enable direct comparisons. Separate two-tailed Mann–Whitney–Wilcoxon tests were performed pairwise between ASD and controls for each microstate class in every temporal parameter to identify statistically significant differences and then corrected for multiple comparisons by applying the FDR.

Table 1 is divided into four major sections to illustrate the results of the microstate analysis. Each division, containing four rows, is labeled with the respective temporal parameter, summarizing the mean values and the standard deviation of every microstate class (the six columns) for the ASD and control groups in the first and second row, respectively; the *p*-value (pairwise) and the corrected *p*-value for multiple comparisons are displayed in the third and fourth row, respectively. The statistically significant differences (*p* < 0.05) in the last two rows are marked with an asterisk. It was observed that microstate classes B, C, and E exhibited significant group differences in some of the temporal parameters after the pairwise comparison, but only classes B and E demonstrated statistical significance at a 5% level after the correction for multiple comparisons.

**Table 1:** Temporal parameters in the microstate analysis of the ASD and control groups.

	Microstate classes					
	
	Class A	Class B	Class C	Class D	Class E	Class F
**Mean duration [milliseconds]**						
ASD (mean ± SD)	76.29 ± 6.08	80.60 ± 4.45	87.16 ± 8.67	77.59 ± 6.94	74.26 ± 5.21	75.17 ± 6.23
Controls (mean ± SD)	78.79 ± 6.54	76.40 ± 7.70	103.35 ± 19.40	74.71 ± 11.76	71.87 ± 10.89	74.18 ± 7.29
*P*-value (pairwise)	0.738	0.077	0.026^∗^	0.410	0.115	0.446
Corrected *P*-value	0.738	0.230	0.156	0.535	0.230	0.535

**Freq. of occurrence [counts/second]**						
ASD (mean ± SD)	1.73 ± 0.36	2.10 ± 0.41	2.24 ± 0.47	1.81 ± 0.38	1.33 ± 0.38	1.58 ± 0.46
Controls (mean ± SD)	1.71 ± 0.43	1.60 ± 0.40	2.54 ± 0.60	1.50 ± 0.64	1.01 ± 0.60	1.49 ± 0.47
*P*-value (pairwise)	0.927	0.008^∗^	0.186	0.131	0.010^∗^	0.522
Corrected *P*-value	0.927	0.030^∗^	0.279	0.262	0.030^∗^	0.626

**Ratio of time coverage**						
ASD (mean ± SD)	0.152 ± 0.043	0.196 ± 0.046	0.232 ± 0.075	0.162 ± 0.046	0.111 ± 0.038	0.138 ± 0.049
Controls (mean ± SD)	0.157 ± 0.050	0.142 ± 0.044	0.345 ± 0.137	0.136 ± 0.083	0.088 ± 0.082	0.128 ± 0.053
*P*-value (pairwise)	0.976	0.021^∗^	0.042^∗^	0.208	0.008^∗^	0.446
Corrected *P*-value	0.976	0.063	0.084	0.312	0.048^∗^	0.535

**Global explained variance (GEV)**						
ASD (mean ± SD)	0.077 ± 0.029	0.102 ± 0.035	0.156 ± 0.076	0.078 ± 0.024	0.055 ± 0.027	0.060 ± 0.026
Controls (mean ± SD)	0.081 ± 0.040	0.064 ± 0.026	0.274 ± 0.140	0.061 ± 0.044	0.038 ± 0.038	0.055 ± 0.029
*P*-value (pairwise)	0.976	0.018^∗^	0.049^∗^	0.131	0.010^∗^	0.483
Corrected *P*-value	0.976	0.054	0.098	0.197	0.054	0.580


*The p-value (pairwise) row corresponds to the result of the pairwise post-hoc Mann–Whitney–Wilcoxon tests. The corrected p-value row was obtained by applying the false discovery rate (FDR) correction for multiple comparisons. Statistically significant differences (p < 0.05) after the tests are marked with an asterisk.*

- Microstate class A did not exhibit significant differences in any of the four temporal parameters (p-pairwise > 0.7; p-corrected > 0.7). However, it was the only class in which the parameters did not display a consistently increased presence in a specific group.
- Microstate class B illustrated a consistently higher presence in ASD, showing statistically significant differences, before or after correction for multiple comparisons, in the frequency of occurrence (p-pairwise = 0.008; p-corrected = 0.030), ratio of time coverage (p-pairwise = 0.021; p-corrected = 0.063), and GEV (p-pairwise = 0.018; p-corrected = 0.054).
- Microstate C was the only class that displayed a consistently higher presence in controls, showing statistically significant differences, before or after correction for multiple comparisons, in the main duration (p-pairwise = 0.026; p-corrected = 0.156), ratio of time coverage (p-pairwise = 0.042; p-corrected = 0.084), and GEV (p-pairwise = 0.049; p-corrected = 0.098). Furthermore, class C was systematically the most dominant in each group.
- Microstate class D exhibited a consistently increased presence in ASD, but without statistically significant differences (p-pairwise > 0.13; p-corrected > 0.26).
- Microstate class E displayed an increased presence in ASD, showing statistically significant differences, before or after correction for multiple comparisons, in the frequency of occurrence (p-pairwise = 0.010; p-corrected = 0.030), ratio of time coverage (p-pairwise = 0.008; p-corrected = 0.048), and GEV (p-pairwise = 0.010; p-corrected = 0.054). Furthermore, class E was systematically the least dominant in each group.
- Microstate class F illustrated a consistently higher presence in ASD, but without statistically significant differences (p-pairwise > 0.44; p-corrected > 0.53).

## Discussion

In this study, we applied the microstate analysis to investigate the differences in four temporal parameters (mean duration, frequency of occurrence, time coverage, and GEV) between 10 autistic and 13 neurotypical young adults in resting state (eyes closed) data. We found that the EEG microstates lasted, on average, for around 75–105 ms, which is in line with the duration reported by different literature reviews (Khanna et al., 2015; Michel and Koenig, 2018).

The two-step cluster analysis combined with the meta-criterion revealed that six template maps best described the entire dataset explaining ∼83% of the global variance. Among these six topographies, it is possible to match the first four maps with the canonical microstates reported in different literature reviews (Khanna et al., 2015; Michel and Koenig, 2018) (classes A, B, C, and D); the other two maps also correspond to classes E and F reported in Bréchet et al. (2019) and Custo et al. (2017). Moreover, considering the four canonical topographies, ∼76% of the global variance is explained. Although predetermining the number of microstates, e.g., to four for the four canonical maps, has shown stable topography maps and is useful to compare or complement results across different studies, we believe that there is not a correct fixed number of classes, and it has been recommended that the number of clusters should be determined specifically for every dataset, based on the explained global variance and functionality, which depends on the conditions within the experiments (Michel and Koenig, 2018).

For the temporal parameters analyzed, microstates C and E were systematically the most and least dominant classes, respectively, during the eyes-closed resting-state analysis. Furthermore, classes B, C, and E exhibited significant group differences in some of the parameters after the pairwise comparison, but only B and E demonstrated statistical significance at a 5% level after the correction for multiple comparisons. However, note that the FDR correction might yield to conservative results, and therefore, the physiological relevance of microstate C is also considered.

Although microstate A did not exhibit any statistically significant difference, interestingly, it was the only class in which the temporal parameters did not display a consistently higher occurrence in a specific group, that is, despite having an increased frequency of occurrence in ASD, the mean duration, GEV, and time coverage were higher in the controls. These results are in line with the study conducted by Jia and Yu (2019), but the authors reported a significant difference in this class. This discrepancy might be mainly due to the key differences between the two studies: age range and the combination of eyes-open and eyes-closed conditions incorporated Jia and Yu (2019). However, according to Tomescu et al. (2018), the development of microstate class A across age does not reveal a statistically significant difference in neurotypical subjects; still, this has not been explored in ASD yet.

Microstate class B displayed a systematically higher presence in the ASD group, being consistent with the results reported in Jia and Yu (2019). Specifically, it illustrated a significant higher frequency of occurrence than the control group. However, the ratio of time coverage and GEV also exhibited statistically significant differences between both groups after the Mann–Whitney–Wilcoxon pairwise tests (see Table 1). Microstate B has been related to the visual network in resting-state (Britz et al., 2010) and verbal processing (Milz et al., 2016). Consequently, the increased frequency of occurrence and time coverage in the ASD might be due to their enhanced inter-network connectivity reported in Morgan et al. (2019). Particularly, the authors reported a significant increased functional connectivity between the language (LAN) and visual (VIS) networks in resting-state fMRI, which is associated with the communication impairment that characterizes ASD. Moreover, a recent study (Bréchet et al., 2019) associates this class with the *scene-reconstruction subsystem*. Therefore, the combination of these findings with the higher presence of this class observed in the ASD group in this work indicates that autistics were more engaged with visual scene-reconstruction memories during resting state.

Microstate class C has a systematically larger occurrence in the temporal parameters for both groups, and it is expected to decrease during visualization (Milz et al., 2016). Additionally, it was the only class that exhibited a higher presence for all the temporal parameters in the control group. However, the significant differences obtained by applying the Mann–Whitney–Wilcoxon pairwise tests in the mean duration, frequency of occurrence, and GEV were not significant at the 5% level after the FDR correction. According to some authors (Michel and Koenig, 2018), microstate class C reflects a portion of the default mode network (DMN), a network where the brain areas involved decrease their activity during attention-demanding tasks (Raichle et al., 2001; Raichle, 2015). This hypothesis is consistent with the observations made by Custo et al. (2017) where the underlying sources associated with microstate C overlap a portion of the DMN, and the significant reduction observed in math conditions (Bréchet et al., 2019) and visualization (Milz et al., 2016) when compared to resting state. The higher presence of microstate class C in the control group might be addressed from the functional perspective. Some studies have found a hypo-connectivity in the posteromedial cortex in ASD (Lynch et al., 2013; Bi et al., 2018), and more importantly, the connectivity within the DMN not only helps in differentiating between ASD and neurotypicals (Assaf et al., 2010; Yao et al., 2016; Morgan et al., 2019) but also might explain the ASD social impairment due to the decreased functional connectivity between the precuneus and medial prefrontal cortex/anterior cingulate cortex (Assaf et al., 2010; Yao et al., 2016), which are regions associated with the resting-state networks in microstate class C (Britz et al., 2010).

A recent study (Bréchet et al., 2019) investigated the resting state compared to conditions of cognitive tasks involving self-related memories (memory condition) and arithmetic calculations (math condition). The study reported that compared to resting state, there is a significant reduction in the incidence of microstate C for the math condition, and no statistically significant difference in the presence of microstate C for memory conditions. Hence, the increased presence of class C in the control group could also imply that the neurotypicals were more focused on self-memory retrieval during the resting state.

Microstate class E was systematically the least dominant in the four temporal parameters analyzed, showing a significant difference between both groups in the frequency of occurrence and time coverage. Very few studies have reported the microstate E presence (Custo et al., 2017; Serrano et al., 2018; Wei et al., 2018; Bréchet et al., 2019), and its functional significance has not been explored. However, the brain regions associated with microstate E reported in Bréchet et al. (2019) and Custo et al. (2017) include the anterior cingulate cortex (ACC), which is also a brain area implicated, among others, in the repetitive behaviors in ASD (Amaral et al., 2008).

The EEG microstates analysis technique is applied over a broad frequency band. The presence and interpretation of microstate classes within narrower frequency bands and the relationship of these states to EEG rhythms, while outside of the scope of the research presented here, are an important topic and a worthy focus of future research to evaluate the impact on the different temporal parameters.

## Conclusion

The main purpose of this study was to compare the EEG resting state between neurotypicals and ASD young adults applying microstate analysis, focusing on the analysis of mean duration, the frequency of occurrence, the ratio of time coverage, and the GEV. The grand-cluster analysis revealed that across all subjects, six template maps best described the complete dataset with ∼83% of the global variance. We suggest that unless a study is aimed to compare or complement previous reports, the number of microstates classes should be selected for each dataset individually, considering the explained global variance and the significance of the classes, depending on the conditions within the experiment.

We performed this study considering resting state only, finding important differences between both groups, and these results should be contemplated as a reference for further assessments comparing the different classes and both groups under task-oriented experiments. Specifically, (1) since microstate class C was the only one that exhibited a consistently increased incidence in controls, it would be interesting to quantify the decreasing presence compared to the ASD group once the subjects start being cognitively engaged, and (2) evaluate if microstate class E is still present under certain types of tasks.

## Data Availability

The datasets generated for this study are available on request to the corresponding author.

## Ethics Statement

The experiment was approved by the Human Subjects Internal Review Board at Texas Tech University. All subjects gave written informed consent in accordance with the Declaration of Helsinki.

## Author Contributions

DD-B and MB contributed to the conception of the study. MB provided the database and provided expertise on autism. DD-B conducted the pre-processing of the data, ran the microstates analysis and statistical tests, and wrote the initial draft of the manuscript. TK provided expertise on digital signal processing and reviewed the pre-processing stage and statistical analysis. MB and CM provided expertise on EEG signal analysis. DD-B and CM provided the functional significance of the microstates. MB, CM, and TK critically revised the different drafts, providing valuable feedback in all of them. All authors contributed to the interpretation of the results and manuscript revision, and read and approved the submitted version.

## Conflict of Interest Statement

The authors declare that the research was conducted in the absence of any commercial or financial relationships that could be construed as a potential conflict of interest.

## Footnotes

1 https://sites.google.com/site/cartoolcommunity/
